# Recent Advances in Radiotracer Imaging Hold Potential for Future Refined Evaluation of Epilepsy in Veterinary Neurology

**DOI:** 10.3389/fvets.2017.00218

**Published:** 2017-12-13

**Authors:** Marion Bankstahl, Jens P. Bankstahl

**Affiliations:** ^1^Department of Pharmacology, Toxicology, and Pharmacy, University of Veterinary Medicine Hannover, Center of Systems Neuroscience Hannover, Hannover, Germany; ^2^Department of Nuclear Medicine, Hannover Medical School, Hannover, Germany

**Keywords:** positron emission tomography, single photon emission computed tomography, imaging, epilepsy, biomarker, positron emission tomography, single photon emission computed tomography

## Abstract

Non-invasive nuclear imaging by positron emission tomography and single photon emission computed tomography has significantly contributed to epileptic focus localization in human neurology for several decades now. Offering functional insight into brain alterations, it is also of particular relevance for epilepsy research. Access to these techniques for veterinary medicine is becoming more and more relevant and has already resulted in first studies in canine patients. In view of the substantial proportion of drug-refractory epileptic dogs and cats, image-guided epileptic focus localization will be a prerequisite for selection of patients for surgical focus resection. Moreover, radiotracer imaging holds potential for a better understanding of the pathophysiology of underlying epilepsy syndromes as well as to forecast disease risk after epileptogenic brain insults. Importantly, recent advances in epilepsy research demonstrate the suitability and value of several novel radiotracers for non-invasive assessment of neuroinflammation, blood–brain barrier alterations, and neurotransmitter systems. It is desirable that veterinary epilepsy patients will also benefit from these promising developments in the medium term. This paper reviews the current use of radiotracer imaging in the veterinary epilepsy patient and suggests possible future directions for the technique.

## Introduction

Molecular radiotracer imaging, including positron emission tomography (PET) and single photon emission computed tomography (SPECT), holds a tremendous potential for diagnostics of brain changes. These methods provide functional insight into the brain, which is difficult to assess by non-invasive techniques. Following injection of a radiolabeled compound, uptake and regional distribution in the brain is three-dimensionally visualized by a PET or SPECT camera based on radioactive decay detection. In addition to radioisotope imaging, up-to-date clinical scanning systems are equipped with fully functional computed tomography (CT). Very recently, integrated magnetic resonance imaging (MRI) components for anatomical co-registration are available. These advances make particularly PET/CT or PET/MRI in combination with various novel radiotracers very promising imaging techniques for evaluation of neurological diseases. Despite the use of radiotracer imaging in human patients for some decades, this imaging modality is only rarely used in clinical veterinary neurology. This is owing to some practical limitations. Due to high cost and radiosafety requirements, PET or SPECT scanners are usually not available in veterinary practices or clinics (1). Usually, only by collaboration with nuclear medicine specialists for human patients, can these techniques be made available for veterinary patients. Clinically used radiotracers have a half-life of minutes to hours and are injected only in nano- to pico-molar concentrations making pharmacological side effects usually negligible. Still, injection of radioisotopes may require hospitalization under a radiosafety regime. Particularly some PET radiotracers with a half-life only up to about 1 h require onsite radiochemistry, often including instant isotope production using a cyclotron. These limitations have led to enormous efforts of radiochemistry research to label PET tracers with fluorine-18 with a half-life of almost 2 h, making delivery from central production sites to imaging centers possible. In spite of these restrictions, the use of radiotracer imaging in veterinary medicine, particularly in veterinary oncology, has increased during the last years. This increase is mostly driven by more individualized tumor diagnostics and treatments (2, 3). In this area, F-18-fluoro-deoxy-glucose (F-18-FDG) is, in analogy to human patients, the most successful radiotracer. F-18-FDG is a radiolabeled glucose analog which is taken up and trapped in metabolically active cells. This feature makes it very useful not only for localization of peripheral and CNS tumors and its metastases but also for measurement of epilepsy-associated altered regional brain activity. In addition to F-18-FDG, in human clinical practice, more and more radiotracers are available targeting, e.g., brain perfusion, neuroreceptor density, brain inflammation, or the burden of amyloid beta in patients with Alzheimer’s disease.

## Role of Radiotracer Imaging in Human Epilepsy

Preliminary findings with newer radiotracers in human epilepsy patients suggest their future potential for disease evaluation and elucidation of pathophysiological mechanisms. However, the clinical application of radiotracer imaging as yet lies mainly on presurgical evaluation of pharmacoresistant patients being considered for focus resection. In these individuals, representing around one-third of epilepsy patients (4), PET and SPECT can be helpful non-invasive tools for identifying the epileptogenic zone. They are of particular value for drug-refractory patients with no structural alterations in MRI, with multifocal MRI-positive lesions which cannot all be assigned to the epileptic focus, or with inconclusive video-electroencephalogram (EEG) monitoring findings (5, 6).

Radiotracers typically used for this purpose target either brain blood perfusion [e.g., Tc-99m-hexamethyl propyleneamine oxime (Tc-99m-HMPAO) SPECT], metabolic pathways, especially glucose metabolism (F-18-FDG PET), or, occasionally, neurotransmitter receptors [e.g., C-11-flumazenil (C-11-FMZ) PET]. Hitherto, F-18-FDG is the most commonly applied tracer for detection of the epileptic focus region and is of high sensitivity particularly in patients with temporal lobe epilepsy (up to 90% sensitivity) (7–9). F-18-FDG PET is usually performed in the seizure-free interval (interictal PET) and aims at identifying brain regions with decreased glucometabolism, being considered to partially reflect, among other factors, reduced synaptic activity (10). F-18-FDG PET can also deliver information about disease severity and progression. In this regard, it has been shown in children with intractable epilepsy that the extent of the hypometabolic brain area can be indicative of the seizure burden, i.e., it grows in size with increasing seizure frequency and *vice versa* (11). Further, F-18-FDG PET can be indicative for occurrence of memory impairment (12, 13) as well as provide prognostic information on seizure freedom after surgery (14).

While most radiotracers targeting metabolic pathways or brain perfusion need an accumulation time much longer than the duration of a single seizure, Tc-99m-HMPAO SPECT, due to the high first-pass uptake of the radiotracer, can be applied during seizure activity (ictal SPECT). Furthermore, the radioactive half-life of Tc-99m of about 6 h enables a stand-by availability of the radiotracer in a video-EEG monitoring unit. To enable diagnostic success, an established setup of continuous video-EEG monitoring for immediate seizure detection of the respective patient and continuous access to the radiotracer for prompt injection immediately after seizure onset is mandatory. As Tc-99m-HMPAO accumulates in areas with high blood flow, the hyperperfused seizure focus displays a distinct increase in tracer signal. Ictal SPECT is associated with a correct focus detection in most patients with temporal lobe epilepsy (>80% sensitivity) (15, 16). In case of additionally performed interictal Tc-99m-HMPAO SPECT, the probability for detecting the seizure focus may increase by applying SISCOM analysis, i.e., subtracting interictal SPECT images from the ictal images and displaying the results on co-registered MR images (5, 17).

Diagnostic evaluation for identifying increased or decreased regional tracer uptake is usually performed by nuclear medicine physicians together with neurologists primarily by visual analysis. In principal, every brain region can be affected, directly but also as a consequence of diaschisis, i.e., secondary functional lesions in brain areas influenced by the primary affected brain region. Regions frequently affected in temporal lobe epilepsy patients include ipsilateral hippocampus, amygdala, thalamus, frontal cortex, and insula (17). Other methods of data evaluation like statistical parametric mapping analysis are not common in current clinical routine, yet can significantly increase the diagnostic sensitivity of combined ictal-interictal Tc-99m-HMPAO SPECT (17, 18).

## Current State in Veterinary Medicine

For several years now, F-18-FDG PET/CT is increasingly used also in veterinary oncology (2, 19). Initial reports indicate that F-18-FDG PET may indeed be useful for localization of brain tumors in dogs (20, 21). Brain tumors are one common MRI-positive cause of structural epilepsy in dogs (22) as well as in human patients (23). In addition, F-18-FDG brain reference data in healthy Beagles have been provided (24).

**Figure 1:**
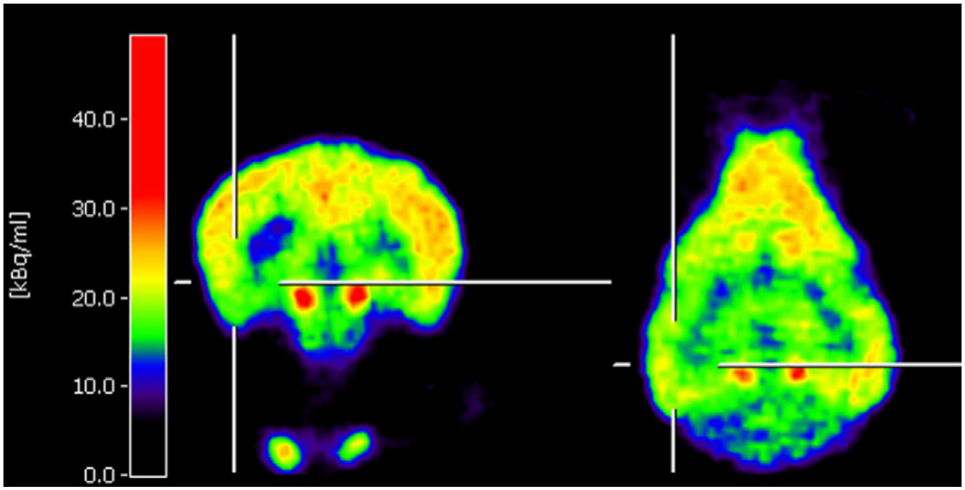
Coronal and horizontal F-18-fluoro-deoxy-glucose positron emission tomography (PET) images from a 10.5-week-old puppy with juvenile epilepsy showing an area of hypometabolism in the left temporal lobe, indicated by the cross localizers. The hot scale represents radioactivity concentration (kBq/ml) [taken from Ref (25), permission from the copyright holder has been obtained].

Neuro-nuclear imaging in dogs and cats may also serve to identify an epileptic focus in MRI-negative epilepsy. Currently, PET or SPECT are barely used for this purpose in veterinary medicine. Nonetheless, joint efforts in Finland have led to two recent publications supporting that F-18-FDG PET for identification of the epileptic focus region is translatable to veterinary patients. In juvenile Lagotto Romagnolo dogs with focal-onset epilepsy, Jokinen and colleagues identified regions with reduced glucose metabolism in cortical brain regions associated with EEG abnormalities (25). Figure 1 shows an interictal F-18-FDG PET image taken from this study displaying glucose hypometabolism in the left temporal lobe. A second study performed by the same group prospectively evaluated adult Finnish Spitz dogs with focal idiopathic epilepsy by EEG and F-18-FDG and found abnormalities by visual analysis in 9/11 dogs with occipital cortex findings most consistent with the epileptic status (26). Although changes in F-18-FDG uptake were also detected by this method in part of the controls of the investigated breed, quantification on group level resulted in statistically significant lower uptake values in epileptic dogs in the hippocampus, cortical regions, and the cerebellum. Considerably, in this regard, PET imaging data have been reported to be of higher diagnostic sensitivity than visual analysis of EEG recording (26). Preferably, further prospective studies with larger group sizes will confirm these findings and evaluate whether they are representative also for other breeds. Of course, studies in epileptic cats are also desirable.

Assessment of brain perfusion by SPECT was first investigated in healthy dogs more than 15 years ago using the Tc-99m-ethyl cysteinate dimer (Tc-99m-ECD) (27). More recently, interictal Tc-99m-ECD SPECT/CT was also explored in dogs with idiopathic epilepsy, reporting hypoperfusion restricted to subcortical areas of epileptic dogs (28). Subsequently, the same group was able to further differentiate subcortical brain regions in healthy dogs, including hippocampus, thalamus, and striatum, using a high resolution SPECT scanner, but did not yet apply this technique to veterinary epilepsy patients (29). Certainly, one has to keep in mind that interictal perfusion SPECT is distinctly less sensitive for seizure focus identification compared to ictal perfusion SPECT (16, 18). Due to the logistic requirements for ictal perfusion SPECT, particularly the availability of a video-EEG monitoring unit in proximity to the scanner, however, this technique has obvious limitations for clinical routine in veterinary neurology. Nonetheless, recent progress in developing devices for non-invasive video-EEG recording (30) or seizure-alert systems based on invasive EEG recording in dogs (31, 32) might pave the way for such efforts in the close future. Ideally, future ictal perfusion SPECT studies would include the clinically more relevant perfusion tracer Tc-99m-HMPAO.

In contrast to human medicine, imaging procedure in veterinary epilepsy patients requires consideration of several distinctive features. First, anesthesia necessary to achieve immobility of the subject for neuroimaging can considerably influence functional brain imaging results (33, 34). Another impact factor is the chronic anti-seizure medication in epilepsy patients. Indeed, phenobarbital, which still represents the most widely applied anti-seizure drug in dogs and cats, seems to reduce brain glucometabolism and hereby also F-18-FDG PET signal in human epilepsy patients (35). A further factor in veterinary medicine is variation in brain and skull anatomy and size of different dog and cat breeds (36, 37) hampering application of reference PET, SPECT, or MRI brain templates for image analysis. In consequence, for reliable image interpretation establishing diverse sets of reference images for different breeds will presumably be necessary.

## Future Directions

Nuclear imaging is currently experiencing broad application to help filling several gaps in epilepsy research: (i) to elucidate the pathophysiological processes underlying epilepsy development and disease progression (38, 39), (ii) to identify predictive biomarkers for stratification of individuals with high risk of disease development after epileptogenic brain insults (38, 40), and (iii) to identify mechanisms of drug resistance (41).

Lasting seizure burden despite state-of-the-art anti-seizure pharmacotherapy remains a serious problem also in epileptic dogs (42, 43). Interest in establishing epilepsy surgery in pharmacoresistant cats and dogs will probably grow. As in human patients, surgical resection of the epileptic focus region may be advantageous in veterinary patients for achieving seizure freedom or control in carefully chosen refractory individuals. For selection of appropriate epilepsy surgery candidates, proper presurgical evaluation needs to be established. MRI is already widely used as a reliable technique to identify brain abnormalities in epileptic companion animals (44). However, in epilepsy cases with negative MRI, application of nuclear imaging, e.g., F-18-FDG PET or Tc-99m-HMPAO SPECT, has a significant potential to provide the necessary data for epileptic focus localization also in animals.

Besides its established benefit for presurgical patient evaluation, neuroimaging of glucose metabolism might also provide hints for ongoing epileptogenesis before clinical seizures occur. In addition, it might offer information on the brain regions involved in epilepsy development and progression. Meanwhile, a whole batch of F-18-FDG PET studies performed in several rodent models of epileptogenesis shows that glucose metabolism decreases in brain regions associated with epilepsy development already during the latency phase, i.e., the time period between the epileptogenic insult and the first clinical seizure (45–48). In combination with other markers, F-18-FDG PET might, therefore, also serve as a prognostic biomarker for an increased risk to develop epilepsy, which is investigated in ongoing studies. An attempt to use F-18-FDG PET as a marker to predict the epileptic outcome in rats showed indeed promising preliminary results (47), but further studies in larger animal groups will be needed to confirm this approach.

An emerging field in epilepsy research is assessment of neuroinflammation as a process present during epileptogenesis as well as in chronic epilepsy (41). Particularly, radioligands of the so-called translocator protein (TSPO; also known as peripheral benzodiazepine receptor) can be utilized to visualize activated microglia, and to a lesser extent, of reactive astrocytes (49). In animal models of epileptogenesis and chronic epilepsy, various radiolabeled TSPO ligands have been evaluated. The newest-generation ligand F-18-GE180 is characterized by a favorable signal-to-noise ratio across species (50). Data gained by F-18-GE180 PET in a rat model of epileptogenesis demonstrates the suitability of TSPO PET to reveal the time course of neuroinflammation during epilepsy development and to identify brain regions involved in this process (51). Further, TSPO PET at disease onset with a different ligand (F-18-PBR111) has recently been shown to have potential of predicting the frequency of later spontaneous recurrent seizures in rats (52). In chronic epileptic rats, increased TSPO signal was found in phenobarbital-unresponsive but not in phenobarbital-responsive individuals, suggesting that TSPO PET might also serve as an indirect marker for pharmacoresistance (53). In parallel, studies in human patients support potential value of TSPO PET for localizing the seizure focus (54, 55).

In close interaction with, or even as one relevant source of neuroinflammation, increased permeability of the blood–brain barrier (BBB) leading to extravasation of blood compounds like albumin is considered to be another crucial factor for epilepsy development (56). Extravasated albumin was found also in brain tissue of human patients with chronic epilepsy (57), suggesting that BBB leakage might also play a role in epilepsy maintenance or progression. *In vivo* imaging approaches to visualize a leaky BBB are based on detection of contrast agents or radiotracers which do not cross the intact BBB. Contrast-enhanced MRI is an established technique to diagnose BBB leakage after epileptogenic insults (e.g., status epilepticus, stroke, or traumatic brain injury) in rodents and human patients (58, 59), but SPECT and PET using the radiotracers Tc-99m-diethylenetriaminepentaacetic acid and Ga-68-DTPA have also been demonstrated suitable for this purpose (58, 60, 61). While the application of gadolinium-based MRI contrast agents is related with safety risks due to compound accumulation in human brain and kidneys (62, 63), administration of nuclear imaging tracers is considered to be safe. Findings in animal models suggest that BBB leakage is highest in brain regions which are also affected by microglial activation during epileptogenesis (58, 59). The role of BBB leakage for epilepsy development in canine and feline epilepsy and the applicability of respective imaging techniques still remain to be assessed.

Nuclear imaging has also proven to be of some value for identification of human drug-refractory epilepsy patients. Drug-refractory epilepsy patients represent a large proportion of patients in both human and veterinary medicine. One mechanism attributed to drug refractoriness in epilepsy is overexpression of the efflux transporters like P-glycoprotein at the BBB, which extrudes anti-seizure drugs back into the blood, therefore resulting in sub-therapeutic drug levels at the site of the epileptic focus (64). Increased P-glycoprotein expression as mechanism of pharmacoresistance has also been suggested for canine patients (65, 66). Both human and veterinary patients affected by this mechanism might profit from alternatively being treated with anti-seizure drugs not being extruded by P-glycoprotein, or with transporter inhibitors or modulators (64, 67, 68). Prerequisite for translation of such therapeutic approaches to the clinical situation would be a diagnostic tool for identification of individuals with actual transporter overactivity. PET with the P-glycoprotein substrate tracer C-11-verapamil was shown to identify increased transporter function at the BBB in a post-status epilepticus model in rodents (69). This preclinical setup was successfully translated to drug-refractory human patients, demonstrating higher P-glycoprotein activity in pharmacoresistant individuals (70).

Besides F-18-FDG PET, the GABA-A receptor ligand 11-C-FMZ, and more recently also 18-F-FMZ, is more and more used for epileptic focus localization (41). In human patients with mesial temporal lobe epilepsy, F-18-FMZ PET can be even advantageous over F-18-FDG as it can result in more circumscribed visualization of altered temporal lobe areas like the hippocampus (71, 72). Recent generation of other radiolabeled receptor ligands like the NMDA glutamate receptor tracer F-18-GE179, will allow to further assess the role of neurotransmitter systems in epileptogenesis and chronic epilepsy (41). Various other ligands targeting neurotransmitter systems including the serotonin, dopamine, cannabinoid, opioid, or acetylcholine system have been investigated in human epilepsy patients (41). Their potential for canine and feline epilepsy patients still needs to be assessed.

## Conclusion

Radiotracer imaging protocols for detecting abnormal glucose metabolism and brain perfusion appear prospective tools for presurgical evaluation of MRI-negative veterinary epilepsy patients in the future. Growing access to nuclear imaging modalities and recent advances in video-EEG monitoring for seizure warning will likely support this development. Evolution of diverse promising radiotracers for epilepsy research, like TSPO and neuroreceptor ligands, opens up new vistas for elucidating the pathophysiology of epileptogenesis and for predicting the risk of disease development in man, including realistic chances of being subsequently translated to veterinary medicine.

## Author Contributions

MB and JPB performed literature search and wrote the manuscript.

## Conflict of Interest Statement

The authors declare that the research was conducted in the absence of any commercial or financial relationships that could be construed as a potential conflict of interest.
